# Hypoxia inducible factor 1α gene (*HIF-1α*) splice variants: potential prognostic biomarkers in breast cancer

**DOI:** 10.1186/1741-7015-8-44

**Published:** 2010-07-12

**Authors:** Jean-Philippe Dales, Nathalie Beaufils, Monique Silvy, Christophe Picard, Vanessa Pauly, Vincent Pradel, Christine Formisano-Tréziny, Pascal Bonnier, Sophie Giusiano, Colette Charpin, Jean Gabert

**Affiliations:** 1 Plateforme Transcriptome, CRO2, Marseille, France; 2 Department of Pathology, Hôpital Nord, Marseille, France; 3 Biochemistry and Molecular Biology, Hôpital Nord, Marseille, France; 4 Etablissement Français du Sang Alpes Méditerranée, Marseille, France; 5 Department of Medical Information, Hôpital Sainte Marguerite, Marseille, France; 6 Department of Gynaecologic Oncology, Hôpital de la Conception, Assistance-Publique Hôpitaux de Marseille, Université de la Méditerranée, Marseille, France

## Abstract

**Background:**

Hypoxia-inducible factor 1 (HIF-1) is a master transcriptional regulator of genes regulating oxygen homeostasis. The HIF-1 protein is composed of two HIF-1α and HIF-1β/aryl hydrocarbon receptor nuclear translocator (ARNT) subunits. The prognostic relevance of HIF-1α protein overexpression has been shown in breast cancer. The impact of HIF-1α alternative splice variant expression on breast cancer prognosis in terms of metastasis risk is not well known.

**Methods:**

Using real-time quantitative reverse transcription PCR assays, we measured mRNA concentrations of total *HIF-1α *and 4 variants in breast tissue specimens in a series of 29 normal tissues or benign lesions (normal/benign) and 53 primary carcinomas. In breast cancers *HIF-1α *splice variant levels were compared to clinicopathological parameters including tumour microvessel density and metastasis-free survival.

**Results:**

*HIF-1α *isoforms containing a three base pairs TAG insertion between exon 1 and exon 2 (designated *HIF-1α*^*TAG*^) and *HIF-1α*^*736 *^mRNAs were found expressed at higher levels in oestrogen receptor (OR)-negative carcinomas compared to normal/benign tissues (*P *= 0.009 and *P *= 0.004 respectively). In breast carcinoma specimens, lymph node status was significantly associated with *HIF-1α*^*TAG *^mRNA levels (*P *= 0.037). Significant statistical association was found between tumour grade and *HIF-1α*^*TAG *^(*P *= 0.048), and total *HIF-1α *(*P *= 0.048) mRNA levels. *HIF-1α*^*TAG *^mRNA levels were also inversely correlated with both oestrogen and progesterone receptor status (*P *= 0.005 and *P *= 0.033 respectively). Univariate analysis showed that high *HIF-1α*^*TAG *^mRNA levels correlated with shortened metastasis free survival (*P *= 0.01).

**Conclusions:**

Our results show for the first time that mRNA expression of a *HIF-1α*^*TAG *^splice variant reflects a stage of breast cancer progression and is associated with a worse prognosis.

See commentary: http://www.biomedcentral.com/1741-7015/8/45

## Background

Hypoxia-inducible factor 1 (HIF-1) is a ubiquitously expressed master transcriptional regulator of many genes regulating mammalian oxygen homeostasis [1,2]. HIF-1 alters transcription of genes mainly involved in energy metabolism, neovascularisation, survival, internal pH and cell migration, and has become recognised as a strong promoter of tumour growth [3,4]. HIF-1 is a heterodimeric protein composed of two HIF-1α and HIF-1β/aryl hydrocarbon receptor nuclear translocator (ARNT) subunits [5]. These subunits are members of the basic helix-loop-helix (bHLH) transcription factor superfamily containing a PAS [PER (Period Clock) -ARNT-SIM (Single-minded)] domain [2]. The HIF-1α subunit is specific to HIF-1 and is the unique oxygen-regulated subunit that determines HIF-1 activity [6,7] whereas HIF-1β is a common subunit for several transcription factors. In human tumours, HIF-1α protein is overexpressed because of intratumoural hypoxia and genetic alterations affecting key oncogenes and tumour suppressor genes [8,9]. This overexpression correlates with tumour angiogenesis, treatment failure and patient mortality [3].

Our group and others have previously reported that HIF-1α protein overexpression is a marker of poor prognosis in primary breast cancer patients [10,11]. HIF-1α is known to be mainly post-transcriptionally regulated by protein ubiquitination and interaction with the Von Hippel-Lindau tumour suppressor protein, and then degraded by the proteasome. Hypoxia stabilises HIF-1α protein by relaxing its ubiquitin-proteasome degradation [12] and affects subcellular localisation, DNA binding capacity and transcriptional activation function of the HIF-1 complex. HIF-1α protein is composed of 826 amino acids [5]. Its N-terminal region contains the bHLH and the PAS domains, which are essential for DNA binding and dimerisation [13]. Its C-terminal region contains two transactivation domains and a nuclear localisation signal [14]. In the middle section of HIF-1α lies a Pro-Ser-Thr oxygen-dependent degradation domain (ODDD, amino acids 401 to 603), which is responsible for the stability of the HIF-1α protein [12].

Although HIF-1α protein regulation has been well studied, *HIF-1α *messenger regulation has only recently begun to be documented [15]. HIF-1α is encoded by the *HIF1A *locus, which is located on human chromosome 14q21-q24 and encodes for 15 exons, resulting in a principal transcript of about 8 kb [16]. Different functional domains of the HIF-1α protein are encoded by separate exons. In addition to the originally described wild type of HIF-1α (HIF-1α^WT^) [1], eight alternative splice variants of *HIF-1α *(HIF-1α^827^, HIF-1α^736^, HIF-1α^557^, HIF-1α^516^, HIF-1α^785^, HIF-1α^417^, HIF-1α^TE ^and an isoform with alternative exon 1 (I.2 isoform)) with different activity have been reported in human cell lines [17-23]. All *HIF-1α *splice variants have been shown to be translated, to dimerise with *HIF-1β *and to be active in human cells. Only one isoform (*HIF-1α*^*736*^) has been documented in human breast cancer [24]. The aim of this study was to analyse *HIF-1α *splice variant expression in human breast cancer and to evaluate its clinical relevance in terms of prognosis. In this study we quantified the mRNA levels of 3 isoforms (*HIF-1α*^*736*^, *HIF-1α*^*557 *^and *HIF-1α*^*516*^) in breast specimens of 53 primary cancers and 29 normal tissues or benign lesions by real-time quantitative reverse transcription PCR (RT-qPCR) assays. *HIF-1α*^*827 *^is similar to wild type except for an additional three base pairs TAG insertion at the exon 1 to 2 junction involving a difference of two amino acids upstream of the bHLH domain [17]. *HIF-1α*^*736 *^is also characterised by an additional three base pairs TAG insertion at the exon 1 to 2 junction, and the lack of exon 14, which produces a frame shift and introduces a stop codon in the coding sequence after the Ile^735 ^[17]. *HIF-1α*^*557 *^loses exon 12 and *HIF-1α*^*516 *^lacks exons 11 and 12 [18,19]. *HIF-1α*^*557 *^has been shown to be induced by zinc ions. *HIF-1α*^*557 *^and *HIF-1α*^*516 *^have been shown to function as dominant negative isoforms of *HIF-1α in vitro*. *HIF-1α*^*785 *^is deprived of exon 11 and was described as being induced *in vitro *by phorbol-12-myristate-13-acetate [20]. *HIF-1α*^*417 *^isoform is deprived of exon 10 [21]. The *HIF-1α*^*TE *^variant with alternative exon 1 (I.1 isoform) was previously found to be expressed in the testis [22]. For these reasons, both these isoforms were thought to be out of the scope of this study on breast tissues samples. We also developed tools to quantify expression of *HIF-1α *splice transcripts characterised by insertion of a three base pairs TAG at the exon 1 to 2 junction (which we designated *HIF-1α*^*TAG*^) involving a difference of two amino acids upstream of the bHLH domain (*HIF-1α*^*827 *^and *HIF-1α*^*736 *^isoforms) [17]. The last known isoform was not quantified because it was described in humans after the end of our study [23]. Expression levels of these isoforms were also compared to standard prognostic clinicopathological parameters, microvessel density of tumours and clinical outcome.

## Methods

### Patients and breast tissue samples

A series of 29 normal tissues or benign lesions (normal/benign) of breast tissue and 53 primary invasive breast carcinomas tissue specimens were selected. All frozen tissues were available in the tumour library of the Department of Pathology (Hôpital Nord, Assitance Publique-Hôpitaux de Marseille, France). Each patient gave informed consent and the study was approved by our institutional review board. Within 15 min of excision fresh tissue samples were frozen in liquid nitrogen by the pathologist (freezing delay shorter than 15 min). All tissue specimens were then stored at -80°C and were never thawed (freezers under temperature control 24 h a day). Specimens of normal breast tissues were obtained from adjacent tumour-free tissue taken from 12 breast cancer patients. Benign lesions were obtained from women undergoing surgery for fibroadenoma (n = 13) or adenosis (n = 4). Normal/benign tissues were obtained from 29 patients aged from 12 to 79 years (mean 44.7 years, SD 16.9). Malignant tumour specimens represented 53 early invasive breast adenocarcinomas diagnosed between 9 September 1989 and 1 May 1996. Tumour tissue specimens were obtained from patients aged from 30 to 84 years (mean 54 years, SD 11.4). Surgery was in all cases the first treatment. The patients underwent axillary node excision combined with wide local excision with margin clearance or mastectomy according to current European recommendations in the department of Oncologic Gynaecology in Hôpital de la Conception, Marseille. For this first step of treatment, patient management was handled by the same group of surgeons (PB). Likewise, radiotherapy, chemotherapy and hormone therapy were applied according to criteria currently used at that time. Each of the respective areas was identified microscopically and dissected immediately upon receipt of the breast specimen by the pathologist. The tumour content of each specimen was verified by histological examination of frozen section by haematoxylin staining. Histological examination of surgical specimens was also performed on paraffin embedded sections stained with haematoxylin, eosin and saffronin. Tumour grading, initially assessed by using the grading methods of Bloom and Richardson [25], was re-evaluated according to Elston and Ellis [26]. Duration of follow-up ranged from 9 to 162 months (mean 80 months, SD 43). For statistical purposes, we selected approximately equal numbers of long (n = 31) and short metastasis-free survival patients (n = 22).

### Clinical and pathological parameters

Lymph node status, tumour size, peritumoural vascular invasion, histological type and histological grade of tumours were obtained from pathological reports. The oestrogen receptor (OR) and progesterone receptor (PgR) status was determined by immunohistochemistry on paraffin-embedded sections as previously described [27,28].

### Tumour microvascular density

Immunodetection studies were performed on frozen sections 5 ?m thick and automated immunoperoxidase procedures as previously reported [29].

### RNA extraction and reverse transcription

Homogenising Trizol procedures have been performed in accordance with current protocols. Frozen tissue (25 to 50 mg) was homogenised in 0.5 ml Trizol reagent (Life Technologies, Carlsbad, California) with lysing matrix D in a FastPrep machine (Q-BIOgene, MP Biomedicals, Illkirch, France) for six cycles of 30 s at a setting of 6 m/s as recommended by the manufacturer. Chloroform (100 μl) was added to each sample, which were mixed vigorously for 15 s and incubated for 3 min at room temperature. Phases were separated by centrifugation (12,000 *g *for 15 min at 4°C) and the aqueous phase was recovered. Isopropanol (250 μl) was added to each of the samples, which were then mixed by inversion and incubated for 10 min at room temperature. Total RNA was pelleted by centrifugation (12,000 *g *for 10 min at 4°C), washed with 1 ml of ice-cold 75% ethanol, air dried and resuspended in 15 μl Rnase-free distilled water. Then, 1 μg of RNA was reverse transcribed with 200 U MMLV Reverse Transcriptase following the Europe Against Cancer (EAC) protocol [30].

### Generation of standard plasmids

pcDNA3 plasmids containing *HIF-1α*^*827 *^and *HIF-1α*^*736 *^sequences with the three base pairs TAG insertion were kindly provided by J Pouyssegur (Institut de Signalisation, Biologie du Développement et Cancer, Centre Lacassagne, Nice, France). For other splice variants, cDNA from human embryonic kidney (HEK)293 cells was amplified using the following primers, respectively localised in exons 1 and 2: forward, 5'-CCGGCGGCGCGAACGACAAG-3', reverse, 5'-TGCGAACTCACATTATGTGG-3', or primers localised in exons 10 and 14, respectively: forward, 5'-TGACCCTGCACTCAATCAAG-3', reverse, 5'-AGTAGCTGCATGTACGTCTG-3'. Conditions for PCR amplification were: 10 min denaturation at 95°C followed by 35 cycles of 30 s at 95°C, 1 min at 55°C, 1 min at 72°C, and the last elongation at 72°C for 10 min. If necessary, DNA bands of interest were isolated and purified from 2% agarose gel and cloned into the pCRII TOPO vector (TOPO-TA cloning kit, Invitrogen, Life Technologies, Carlsbad, California) according to the manufacturers. Plasmids were purified (Nucleospin plasmid, Macherey Nagel, Hoerdt, France) and the cloned inserts were sequenced. Serial dilutions were prepared in *Escherichia coli *rRNA (Roche Diagnostics, Meylan, France) to generate RT-qPCR standard curves (PCT international application no. 02/00937 of 15 March 2002, in the name of Université de la Méditerranée) according to the EAC strategy [30].

### Design

**Figure 1:**
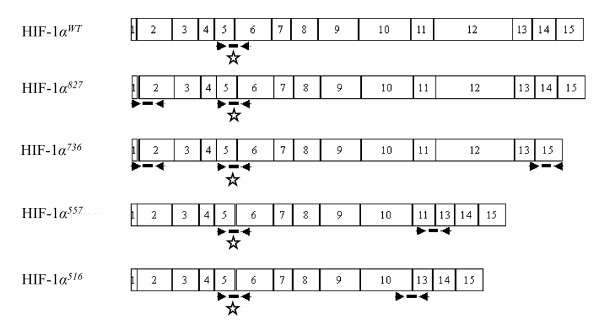
**Position of primers and oligonucleotide probes designed to amplify hypoxia inducible factor 1α (*HIF-1α*) mRNA splice variants**. The diagram illustrates the combination of primers and probes used to detect mRNA of four alternative spliced isoforms of *HIF-1α*. Boxes represent exons designated by 1 to 15. Primers are indicated by solid arrowhead and probe by thick solid lines. Wild-type *HIF-1α *(*HIF-1α*^*wt*^) mRNA consists of 15 exons and 14 introns. Variants reported here are generated by a TAG insertion between exon 1 and exon 2 (*HIF-1α*^*827 *^and *HIF-1α*^*736*^), exon 13 to 15 alternative splicing (*HIF-1α*^*736*^), exon 11 to 13 alternative splicing (*HIF-1α*^*557*^) and exon 10 to 13 alternative splicing (*HIF-1α*^*516*^). Primers on exons 5 and 6 (asterisk) are suitable for quantification of all *HIF-1α *transcripts including *HIF-1α*^*wt *^as well as each isoform. Primers pairs on exons 13 and 15, 11 and 13, 10 and 13 are suitable for specific detection of *HIF-1α*^*736*^, *HIF-1α*^*557 *^and *HIF-1α*^*516 *^variants respectively. Note that primers on exons 1 and 2 allow mRNA detection of both isoforms *HIF-1α*^*827 *^and *HIF-1α*^*736*^.

mRNA sequences of four spliced variants (*HIF-1α*^*TAG*^, *HIF-1α*^*736*^, *HIF-1α*^*557 *^and *HIF-1α*^*516*^) were constructed using the human *HIF-1α *transcript sequence NM_001530 as a reference. Primer and probe sets were designed using the Primer Express software (Applied Biosystems, Courtaboeuf, France) and chosen based on their exon-exon junction location (Figure 1). *HIF-1α*^*736*^, *HIF-1α*^*557 *^and *HIF-1α*^*516 *^splice variant specific TaqMan assays, and an assay specific to a common region present in all known variants and located at exons 5 and 6, were designed. Specific probes and primers on adjacent exons were also designed to detect *HIF-1α *transcripts containing a three base pairs TAG insertion between exon 1 and exon 2 (which we designated *HIF-1α*^*TAG*^). For this variant, primers and probes were designed to detect intron length between exons rather than exon-exon junctions. The length of introns between these exons on DNA is long enough to avoid DNA amplification.

### Optimisation of RT-qPCR

Amplification and quantification of HIF-1α variants was performed on a MX3000P system (Stratagene, Agilent Technologies, Santa Clara, California). Amplifications were performed on specific plasmid serial dilutions of each variant in a total volume of 25 μl containing 1 × TaqMan universal PCR MasterMix (Applied Biosystems, Courtaboeuf, France) and both forward and reverse primers and probes. Different ranges of primer concentrations were tested and optimised for each amplicon to obtain the most specific, sensitive and efficient RT-PCR amplification. Selected primer and probe concentrations are shown in Table 1. Optimised RT-qPCR reaction conditions for gene amplification were as follows: 50°C for 2 min, denaturation for 10 min at 95°C followed by 50 cycles of 30 s at 95°C, 1 min annealing and extension at 60°C for all transcripts except for *HIF-1α*^*TAG *^with 1 min at 62°C. For validation of assays specificity, the primers and probes used to detect each splice variant were tested by RT-PCR with 1 μg RNA of the HEK293 cell line (DSMZ), 1 μg genomic DNA of peripheral blood mononuclear cells (PBMCs) and standards (10^6 ^copies of plasmids) specific for another splice variant. No template control (NTC) was also performed. RT-qPCR products were analysed by Agilent 2100 Electrophoresis Bioanalyzer (Agilent Technologies, Santa Clara, California). Serial dilutions of specific standard plasmids were used to validate sensitivity, linearity and detection limits of RT-qPCR assays. Intrareproducibility and inter-reproducibility assays were performed by four RT-qPCR experiments, each time in triplicate.

**Table 1:** Details of oligonucleotide sequences of primers and probes used to quantify hypoxia inducible factor 1α (HIF-1α) mRNA splice variants by real-time quantitative reverse transcription PCR assays

Oligonucleotide	Position (exon)	5' Position	Sequence (5' to 3')	Primer/probe concentrations (nM)	Amplicon size
	5	518	F: GTACCCTAACTAGCCGAGGAAGAA	300	
		
	6	597	R: GTGAATGTGGCCTGTGCAGT	300	
		
*HIF-1α *(total)	5 to 6 junction	544	P: ATGAACATAAAGTCTGCAACATGGAAGGTATTG	200	80 bp

	1	17	F: GCGCGAACGACAAGAAAAATAG	50	
		
	2	138	R: TGGCAACTGATGAGCAAGCT	300	
		
*HIF-1α*^*TAG*^	2	74	P: ATGCAGCCAGATCTCGGCGAAGTAAA	200	122 bp

	13 to 15 junction	2,182	F: TCACTTTTTCAAGCAGTAGGAATTATTTAG	600	
		
	15	2,330	R: TAATTCTTCACCCTGCAGTAGGTTT	600	
		
*HIF-1α*^*736*^	15	2,260	P: TGACCAGTTATGATTGTGAAGTTAATGCTCCTATACAAGG	200	149 bp

	11	1,566	F: TGTGGATAGTGATATGGTCAATGAATT	300	
		
	13	1,699	R: TAGTATCTTTGGATTTAGTTCTTCCTCAGG	300	
		
*HIF-1α*^*557*^	11 to 13 junction	1,642	P: AAGCAAACCCATTTTCTACTCAGAACTACA	200	134 bp

	10	1,490	F: AGACACCTAGTCCTTCCGATGG	600	
		
	13	1,580	R: AAGCTAGTATCTTTGGATTTAGTTCTTCCT	600	
		
*HIF-1α*^*516*^	10 to 13 junction	1,513	P: AGCACTAGACAAAGTTCACCTGAGAACTACAGT	200	91 bp

The mRNA sequences of four alternatively spliced variants (*HIF-1α*^*TAG*^, *HIF-1α*^*736*^, *HIF-1α*^*557 *^and *HIF-1α*^*516*^) were constructed using the human *HIF-1α *transcript sequence NM_001530. bp = base pairs; F = forward primer; P = oligonucleotide probe; R = reverse primer.

### RT-qPCR assays

Amplifications were performed with 5 μl of cDNA of each sample using RT-qPCR optimised conditions as previously described. mRNA from HEK293 cells was used as positive control throughout the study. Standard (10^6 ^copies of plasmid) specifics of another splice variant were amplified as negative control throughout the study and NTC was performed. Standard curves were generated by serial dilutions of HIF-1α splice variant plasmids ranging from 1 to 10^6 ^copies and used for calculation of copy numbers (CN) for each transcript. Transcripts of the gene coding for the TATA box-binding protein (TPB) were also quantified as endogenous gene controls, and each sample was normalised on the basis of its TBP content as previously described [31]. All experiments were carried out with duplicates for each data point. For each experiment sample, the mRNA copy number of *HIF-1α *gene (*CN*_*HIF-1α*_) and endogenous reference TBP gene (*CN*_*TBP*_) were quantified from standard curves. Final *HIF-1α *mRNA concentrations were expressed in normalised copy numbers (*NCN*_*HIF-1α*_), as follows: *NCN*_*HIF-1α *_= *CN*_*HIF-1α*_/*CN*_*TBP*_.

### Statistical analysis

Differences among groups of breast tissue specimens (normal/benign tissues vs carcinomas) in terms of their *HIF-1α *mRNA levels were assessed using Kruskal-Wallis and Mann-Whitney tests with Bonferroni correction. Since levels of expression in breast cancer specimens showed non-Gaussian distribution, non-parametric tests were used for the analysis of correlation with clinicopathological parameters. Metastasis-free survival (MFS) time, defined as the time from surgery until diagnosis of metastasis, was used as a follow-up endpoint. Regression models (univariate and multivariate) were used to compare survival and identify predictors of survival. All preoperative predictors (clinicopathological and biological parameters) were included in the analysis. The predictors with *P *value < 0.20 in the univariate model were tested in the multivariate regression model. Then backward conditional method was used for variable selection by the Cox multivariate regression model. Finally, variables with adjusted *P *values < 0.05 were kept into the final model. All statistical analyses were performed with SPSS V.13.0.1 software (SPSS, Chicago, IL, USA).

## Results

### Clinicopathological parameters

Tumour characteristics are summarised in Table 2. Tumours corresponded to ductal carcinomas (n = 41) and lobular carcinomas (n = 12). Tumours were grade 1 in 9 cases, grade 2 in 26 cases and grade 3 in 17 cases. A mean of 16.9 (SD ± 5.1) lymph nodes were found in axillary node excision, and 22 patients were node positive. All patients underwent axillary node excision combined with wide local excision with margins clearance or mastectomy in the same department. Treatment post surgery consisted of radiotherapy, chemotherapy and hormone therapy performed by the same group of oncologists. Duration of follow-up ranged from 9 to 162 months. The 2004 records showed that among 53 patients, 22 (41.5%) patients relapsed, among whom 18 died.

**Table 2:** Distribution of clinical and histopathological characteristics of breast cancer patients

Parameter	No. of patients	No. of patients with metastasis
Age, years:		
≤ 50	21	8
> 50	30	13
Positive lymph node:		
0	25	6
1 to 3	11	9
4 to 10	4	3
> 10	7	3
Pathological tumour size, mm:		
≤ 10	3	1
11 to 20	25	8
21 to 50	23	12
> 50	2	1
Tumour grade:		
1	9	1
2	26	13
3	17	8
Histological type:		
Tubular	41	18
Lobular	12	4
Peritumoural vascular invasion:		
Absent	12	4
Present	40	18
OR status:		
OR positive	32	10
OR negative	16	10
PgR status:		
PgR positive	26	6
PgR negative	24	16

Because of missing data, the numbers do not always add up to 53.

OR = oestrogen receptor; PgR = progesterone receptor.

### Optimisation and validation of RT-PCR assays

Primers and probes sets for *HIF-1α *variants were tested using RT-qPCR on the HEK293 RNA and specific standard plasmids. Primer concentration optimisation assays were carried out to determine the optimal concentrations for each primer and probe set. To test the specificity and crossreaction of the primers used for RT-qPCR, experiments were performed using genomic DNA, standard plasmids specific of another splice variant, and nNTC. No amplification was observed with genomic DNA of PBMCs, specific standard plasmids of other splice variants and NTC. Serial dilutions of *HIF-1α *variants specific standard plasmid were used to generate a standard curve to assess the intra-assay and interassay reproducibility and sensitivity. For each variant, excellent reproducibility and linearity of standard curves were found. All runs exhibited amplification slopes between -3.31 and -3.55, which correspond to efficiencies higher than 92% and coefficients of correlation between 0.993 and 1. The assays sensitivity was found at 10 copies for all variants. We did not succeed in quantifying the *HIF-1α*^*417 *^isoform.

### Differential expression of *HIF-1α *splice variants in normal/benign and malignant breast tissues

**Figure 2:**
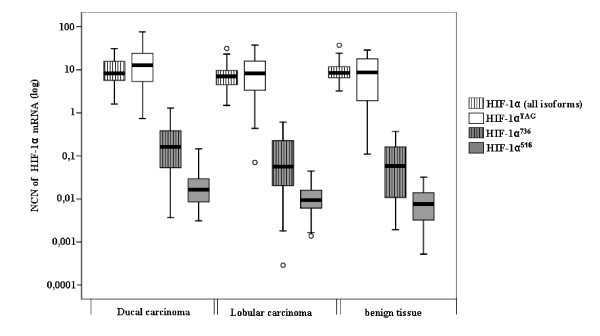
**Distribution of hypoxia inducible factor 1α (*HIF-1α*) mRNA levels in 82 breast tissues determined by real-time quantitative reverse transcription PCR assa*y***. *HIF-1α *mRNA levels were expressed in normalised copy numbers (NCN) on the basis of TATA box-binding protein (TPB) gene content of the tissues as described in the Methods section. Patients grouped according to histological type of tissues (x axis). *HIF-1α *mRNA levels (y axis) were represented according to these different groups of patients. Results were plotted on a logarithmic scale. *HIF-1α *splice variants were detectable in all samples at varying levels. Splice variant *HIF-1α*^*736 *^and *HIF-1α*^*516 *^mRNAs were expressed at levels 100-fold lower than *HIF-1α*^*TAG*^. Variant *HIF-1α*^*557 *^mRNAs that were expressed at levels 1,000-fold lower than *HIF-1α*^*TAG *^are not shown.

**Figure 3:**
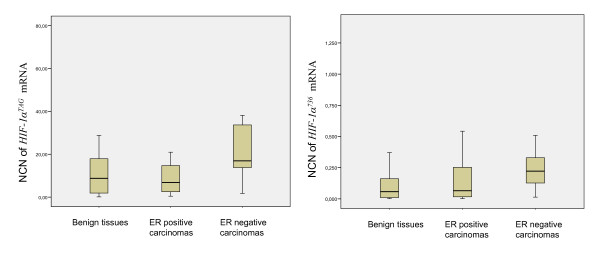
**Distribution of hypoxia inducible factor 1α (*HIF-1α*)^*TAG *^and HIF-1α^736 ^mRNA levels in 82 breast tissues determined by real-time quantitative reverse transcription PCR assay**. *HIF-1α *mRNA levels were expressed in normalised copy numbers (NCN) on the basis of TATA box-binding protein (TPB) gene content of the tissues as described in the Methods section. Patients grouped according to histological type of tissues (x axis). *HIF-1α*^*TAG *^and *HIF-1α*^*736 *^mRNAs were expressed at higher levels in oestrogen receptor (OR)-negative carcinomas compared to normal/benign tissues (*P *= 0.009 and *P *= 0.004 respectively). *HIF-1α*^*TAG *^mRNAs were also higher in OR-negative carcinomas compared to OR-positive ones (*P *= 0.005).

Expression levels of *HIF-1α *mRNAs were determined in each of the 82 samples of breast tissue. *HIF-1α *splice variants were detected in every sample of normal/benign and malignant breast tissues at varying levels. *HIF-1α*^*TAG *^mRNA levels were higher than the other *HIF-1α *splice variants in the majority of breast tissues, and low levels were only found for *HIF-1α*^*736*^, *HIF-1α*^*516 *^and *HIF-1α*^*557 *^isoforms (Figure 2). *HIF-1α*^*TAG *^mRNAs were expressed at similar levels to the total *HIF-1α *expression suggesting that unknown isoforms devoid of exons 5 and 6 may contain the three base pairs TAG insertion. Splice variants *HIF-1α*^*736 *^and *HIF-1α*^*516 *^mRNAs were expressed 100-fold lower than *HIF-1α*^*TAG *^and variant *HIF-1α*^*557 *^mRNA was expressed at levels 1,000-fold lower than *HIF-1α*^*TAG*^. Expression levels of *HIF-1α*^*516 *^and *HIF-1α*^*557 *^mRNA that were very low (mean normalised copy numbers < 0.1) were not included in statistical analyses. There was no significant statistical difference in the total *HIF-1α *expression between normal/benign and malignant breast tissues. *HIF-1α*^*TAG *^mRNAs were also expressed at similar level between the two categories of tissues. *HIF-1α*^*736 *^mRNAs showed a link with malignant phenotype but did not reach statistical significance (*P *= 0.053). Interestingly, *HIF-1α*^*TAG *^and *HIF-1α*^*736 *^mRNAs were found to be expressed at higher levels in OR-negative carcinomas compared to normal/benign tissues (*P *= 0.009 and *P *= 0.004 respectively) (Figure 3). *HIF-1α*^*TAG *^mRNAs were also higher in OR-negative carcinomas compared to OR-positive ones (*P *= 0.005).

### *HIF-1α *splice variants expression correlates with prognostic clinicopathological parameters of breast cancer

Levels of each *HIF-1α *variant mRNA were determined in tumour samples from 53 patients with breast cancer and were compared with lymph node status, tumour size, tumour grade, peritumoural vascular invasion, OR and PgR status. They were also compared with microvessel density of tumours (Table 3). Lymph node status was significantly associated with *HIF-1α*^*TAG *^mRNA levels (*P *= 0.037). Statistically significant association was found between tumour grade and total *HIF-1α *(*P *= 0.048) or *HIF-1α*^*TAG *^(*P *= 0.048). Peritumoural vascular invasion correlated with total *HIF-1α *(*P *= 0.028). Interestingly *HIF-1α*^*TAG *^mRNA levels inversely correlated with both OR and PgR status. No significant association was found between any of the *HIF-1α *mRNA levels and tumour size or microvessel density.

**Table 3:** Relationship between the hypoxia inducible factor 1α (*HIF-1α*) splice variant's expression levels (normalised copy numbers) and clinicopathological factors or microvessel density in tumour tissue specimens from 53 breast cancer patients

Variable	N	Total *HIF-1α*, mean (SD)	*P *value	*HIF-1α*^*TAG*^, mean (SD)	*P *value	*HIF-1α*^*736*^, mean (SD)	*P *value
Lymph node status:							
Negative	25	10.0 (9.0)	NS	10.9 (10.9)	0.037	0.19 (0.29)	NS
Positive	22	12.8 (7.8)		21.1 (19.2)		0.26 (0.28)	
Pathological tumour size:							
< 20 mm	28	10.2 (8.3)	NS	12.4 (13.2)	NS	0.17 (0.26)	NS
≥ 20 mm	25	11.8 (8.3)		17.9 (17.4)		0.25 (0.28)	
Tumour grade:							
1/2 vs	35	9.2 (7.7)	0.048	11.8 (12.3)	0.048	0.19 (0.30)	NS
3	17	14.6 (8.7)		21.5 (19.4)		0.23 (0.20)	
Peritumoural vascular invasion:							
Absent	12	8 (9)	0.028	9.6 (10.1)	NS	0.19 (0.36)	NS
Present	40	11.9 (8.1)		16.6 (16.6)		0.20 (0.24)	
OR status:							
Negative	16	14.2 (7.3)	0.024	20.8 (11.9)	0.005	0.25 (0.19)	0.06
Positive	32	10 (8.8)		12.7 (17.1)		0.20 (0.31)	
PgR status:							
Negative	24	11.8 (7.3)	NS	20.08 (17.8)	0.033	0.23 (0.19)	0.077
Positive	26	10.8 (9.4)		11.3 (12.3)		0.2 (0.34)	
Tumour microvessel density (low vs high)			NS		NS		NS

Because of missing data, the numbers do not always add up to 53. Expression levels of *HIF-1α*^*516 *^and *HIF-1α*^*557 *^mRNA were not included in statistical analyses because of their very low levels (mean normalised copy numbers < 0.1).

NS = non-significant; OR = oestrogen receptor; PgR = progesterone receptor.

### Expression of *HIF-1α*^*TAG *^splice variant correlates with patient survival

**Figure 4:**
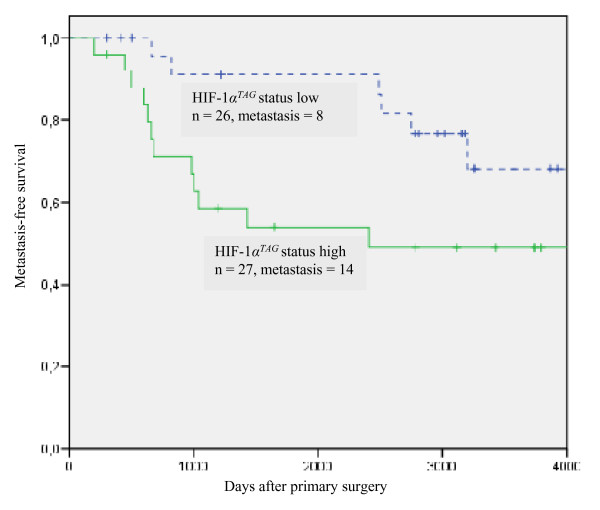
**Kaplan-Meier plot for breast cancer patients illustrating metastasis-free survival according to hypoxia inducible factor 1α (*HIF-1α*)^*TAG *^mRNA levels in breast cancer patients (n = 53)**. Patients with high expression levels of *HIF-1α*^*TAG *^mRNA (median cut-off = 9.95) had a significantly higher risk of relapse (*P *= 0.01).

The strength of association between clinicopathological tumour characteristics (lymph node status, tumour size, tumour grade, peritumoural invasion, OR and PgR status, tumour microvessel density) and expression levels of *HIF-1α *mRNAs with metastasis-free survival is shown in Table 4. Lymph node status (*P *= 0.003), OR status (*P *= 0.021), PgR status (*P *= 0.005) and *HIF-1α*^*TAG *^mRNA levels (*P *= 0.01) were found to be significantly predictive of metastasis free survival. HIF-1α^TAG ^mRNAs were expressed at varying levels. In our series, we used the median value (9.95) of these levels as a cut off to define two groups of patients (the group with mRNA levels higher than 9.95 was considered as a high level of expression and the group with mRNA levels lower than 9.95 was considered as a low level of expression) to evaluate their association with metastasis survival. Breast cancer patients with high expression levels of *HIF-1α*^*TAG *^mRNA had a significantly higher risk of relapse (Figure 4). Expression levels of the other *HIF-1α *transcripts were not associated with prognosis (not shown). Multivariate Cox analysis identified lymph node status (*P *= 0.005) and PgR status (*P *= 0.012) as independent predictors of metastasis free survival. *HIF-1α*^*TAG *^mRNA levels did not remain a significant independent prognostic variable.

**Table 4:** Univariate and multivariate (backward conditional selection) Cox regression analysis of metastasis-free survival (MFS) in breast cancer patients considering clinicopathological characteristics, tumour microvessel density and hypoxia inducible factor 1α (*HIF-1α*)^*TAG *^mRNA levels

Characteristics	Univariate analysis of MFS, Exp B (95% CI)	*P *value	Multivariate analysis of MFS, Exp B (95% CI)	*P *value
Age, years:				
< 50	1	0.561		
≥ 50	1.32 (0.52 to 3.37)			
Positive lymph node:				
0	1	0.003	1	0.005
1 to 3	8.00 (2.56 to 24.81)		5.56 (1.75 to 17.63)	
4 to 9	7.50 (1.70 to 30.00)		6.68 (1.48 to 30.14)	
> 10	1.20 (0.14 to 10.00)		0.48 (0.05 to 4.25)	
Pathological tumour size, mm:				
< 10	1	0.39		
10 to 20	1.10 (0.10 to 8.70)			
21 to 49	2.40 (0.30 to 18.90)			
> 50	2.70 (0.20 to 42.90)			
Tumour grade:				
1/2 vs	1	0.24		
3	1.70 (0.70 to 4.40)			
Peritumoural vascular invasion:				
Absent	1	0.104		
Present	3.40 (0.80 to 14.80)			
OR status:				
Positive				
Negative	0.33 (0.13 to 0.85)	0.021		
PgR status:				
Positive				
Negative	0.20 (0.07 to 0.60)	0.005	0.23 (0.07 to 0.72)	0.012
Tumour microvessel density	1.02 (0.07 to 0.60)	0.22		
*HIF-1α*^*TAG *^mRNA levels	1.03 (1.00 to 1.05)	0.01		

OR = oestrogen receptor; PgR = progesterone receptor, Exp B = exponential of beta

## Discussion

Several isoforms of HIF-1α resulting from alternative splicing have been described in various human tumour cell lines or tissues. Only one isoform (*HIF-1α*^*736*^) has been documented in human breast cancer [24]. Moreover, only a few studies have been performed to link *HIF-1α *mRNA expression and tumour-specific parameters [32,33]. Specific antibodies allowing *HIF-1α *splice variant detection at the protein level are not currently available. We used a real-time quantitative reverse transcription PCR assay to measure mRNA expression levels of 4 alternatively spliced transcripts of *HIF-1α *in breast specimens of 53 primary cancers and 29 normal tissues or benign lesions.

Interestingly, this work shows for the first time higher expression levels of two *HIF-1α *splice variants (*HIF-1α*^*TAG *^and *HIF-1α*^*736*^) in OR-negative carcinomas compared to normal/benign tissues. We also investigated the prognostic value of *HIF-1α *transcript expression levels in breast cancer and found a significant relationship between either clinicopathological characteristics or patient metastasis-free survival. First, we found that *HIF-1α*^*TAG *^mRNAs levels were substantially higher in high grade and steroid hormone receptor-negative tumours. The second and most striking observation was that *HIF-1α*^*TAG *^mRNA levels were indicative of shorter metastasis-free survival, and that this correlated with lymph node status. Contrary to the Cayre *et al*. report [24], we did not find significant correlation between total *HIF-1α *mRNA expression and lymph node status but we observed significant association with tumour grade. This could be because our series was smaller than series of Cayre *et al*. or because the technology used in our study was more sensitive. In our series, total *HIF-1α *mRNA expression negatively correlated with OR status. Similar to Cayre *et al*. we did not find any correlation between total *HIF-1α *mRNA expression and outcome. Our results showing that *HIF-1α*^*736 *^mRNA expression does not correlate with clinicopathological characteristics of tumours also concur with earlier findings of the same group [24]. In this study, effect of adjuvant (none, chemotherapy or hormone) treatment on survival and interactions with expression levels of *HIF-1α *splice variants were not checked because of the limited number of patients in each subgroup. Further experiments in a larger series are required to answer this question.

Alternative splicing is known to play an important role in gene expression regulation by modulating the functional properties of transcription factors [34]. In this regard, alternative splicing can modify DNA-binding properties of transcription factors [35], introduce or eliminate activating domains or increase the *in vivo *stability of a given isoform [36]. Moreover, the abundance of specific isoforms is likely to result from differential expression, RNA stability and selective splicing process leading to an increase of some mRNA species. Recent evidence indicates that in several cancers the ratio of splice variants is dramatically altered and that differential expression of alternatively spliced isoforms in cancer patients can have severe implications for clinical outcome [37]. Remarkably, statistical association has previously been reported between *HIF-1β *splicing variant expression, oestrogen receptors and breast cancer survival [38]. It should be noted that the primers designed on exons 1 and 2 in our study allowed quantification of the *HIF-1α*^*TAG *^sequence present in both *HIF-1α*^*827 *^and *HIF-1α*^*736 *^splice variants [17]. The *HIF-1α*^*TAG *^transcript is characterised by insertion of a three base pairs TAG insertion that may be generated by the use of two potential splice acceptor dinucleotides (AG) of intron 1 at a splice junction site as previously described [39,1]. This splicing results in the replacement of Lys^12 ^by Asn^12 ^and the addition of Arg^13 ^residue located upstream from the bHLH domain of the protein. This replacement may modify the DNA binding affinity of the protein complex as previously shown for Arg^14 ^and Arg^15 ^residues in aryl hydrocarbon receptor (AHR)/aryl hydrocarbon receptor nuclear translocator (ARNT) heterodimer [40]. Further biochemical and structural studies are required for a better understanding of the functional properties of this variant.

## Conclusions

To our knowledge, our results suggest for the first time that at least one *HIF-1α *splice *α *variant may be a marker for the advanced clinical and oestrogen-resistant stage of breast cancer. Based on their correlation with survival, *HIF-1α*^*TAG *^mRNA levels may be a potential useful prognostic indicator whose value should be further validated in prospective studies.

## Competing interests

The authors declare that they have no competing interests.

## Authors' contributions

NB, MS and CP carried out the molecular genetic studies, participated in the sequence alignment and drafted the manuscript. CF participated in the sequence alignment. CC, SG analysed the immunohistochemistry results. VP and VP performed the statistical analysis. J-PD and JG conceived of the study, and participated in its design and coordination. All authors read and approved the final manuscript.

## Pre-publication history

The pre-publication history for this paper can be accessed here:

http://www.biomedcentral.com/1741-7015/8/44/prepub
